# Homocysteine thiolactone and *N*-homocysteinylated protein induce pro-atherogenic changes in gene expression in human vascular endothelial cells

**DOI:** 10.1007/s00726-015-1956-7

**Published:** 2015-03-24

**Authors:** Dorota Gurda, Luiza Handschuh, Weronika Kotkowiak, Hieronim Jakubowski

**Affiliations:** 1 Institute of Bioorganic Chemistry, Poznań, Poland; 2 Department of Biochemistry and Biotechnology, University of Life Sciences, Poznań, Poland; 3 Department of Microbiology, Biochemistry and Molecular Genetics, Rutgers-New Jersey Medical School, International Center for Public Health, 225 Warren Street, Newark, NJ 07101-1709 USA

**Keywords:** Endothelial cells, Homocysteine metabolites, Gene expression, Microarrays, Bioinformatic analysis

## Abstract

Genetic or nutritional deficiencies in homocysteine (Hcy) metabolism lead to hyperhomocysteinemia (HHcy) and cause endothelial dysfunction, a hallmark of atherosclerosis. In addition to Hcy, related metabolites accumulate in HHcy but their role in endothelial dysfunction is unknown. Here, we examine how Hcy-thiolactone, *N*-Hcy-protein, and Hcy affect gene expression and molecular pathways in human umbilical vein endothelial cells. We used microarray technology, real-time quantitative polymerase chain reaction, and bioinformatic analysis with PANTHER, DAVID, and Ingenuity Pathway Analysis (IPA) resources. We identified 47, 113, and 30 mRNAs regulated by *N*-Hcy-protein, Hcy-thiolactone, and Hcy, respectively, and found that each metabolite induced a unique pattern of gene expression. Top molecular pathways affected by Hcy-thiolactone were chromatin organization, one-carbon metabolism, and lipid-related processes [−log(*P* value) = 20–31]. Top pathways affected by *N*-Hcy-protein and Hcy were blood coagulation, sulfur amino acid metabolism, and lipid metabolism [−log(*P* value)] = 4–11; also affected by Hcy-thiolactone, [−log(*P* value) = 8–14]. Top disease related to Hcy-thiolactone, *N*-Hcy-protein, and Hcy was ‘atherosclerosis, coronary heart disease’ [−log(*P* value) = 9–16]. Top-scored biological networks affected by Hcy-thiolactone (score = 34–40) were cardiovascular disease and function; those affected by *N*-Hcy-protein (score = 24–35) were ‘small molecule biochemistry, neurological disease,’ and ‘cardiovascular system development and function’; and those affected by Hcy (score = 25–37) were ‘amino acid metabolism, lipid metabolism,’ ‘cellular movement, and cardiovascular and nervous system development and function.’ These results indicate that each Hcy metabolite uniquely modulates gene expression in pathways important for vascular homeostasis and identify new genes and pathways that are linked to HHcy-induced endothelial dysfunction and vascular disease.

## Introduction

Homocysteine (Hcy) is an important intermediate in one-carbon metabolism that involves the conversion of methionine to cysteine (Mudd et al. 2001) and folate interconversions (Strauss et al. 2007). Deficiencies in these pathways lead to the accumulation of Hcy and its metabolites in the blood—hyperhomocysteinemia (HHcy)—which is an independent risk factor for cardiovascular disease (CVD) and causes endothelial dysfunction, a hallmark of atherosclerosis (Dayal and Lentz 2008). However, molecular mechanisms underlying the pathophysiology of HHcy are not fully understood (Jakubowski 2011, 2013; Perla-Kajan et al. 2007). One hypothesis states that metabolic conversion of Hcy to Hcy-thiolactone initiates a pathway that leads to pathologies associated with HHcy (Jakubowski 1997a, 1999, 2007). Hcy-thiolactone is chemically reactive and modifies ε-amino groups of protein lysine residues, which generates immunogenic and toxic *N*-homocysteinylated protein (*N*-Hcy-protein) (Jakubowski 2008, 2013; Jakubowski et al. 2000).

In humans and mice, HHcy leads to the accumulation of Hcy-thiolactone and *N*-Hcy-protein, in addition to Hcy (Chwatko et al. 2007; Jakubowski et al. 2008, 2009). We and other investigators have shown that HHcy induces changes in gene expression in mouse models that are associated with atherothrombotic disease (Devlin et al. 2005; DiBello et al. 2010; Ingrosso et al. 2003; Kim et al. 2011; Pogribny et al. 2008; Sharma et al. 2006; Suszynska-Zajczyk et al. 2014a, b, c, d). However, it is not known what mechanism(s) are involved and which metabolite—Hcy itself, Hcy-thiolactone, or *N*-Hcy-protein—is responsible for changes in gene expression.

The key to understanding mechanisms by which HHcy disrupts normal cellular function and ultimately causes disease is to identify genes whose expression is affected by individual Hcy metabolites. It is likely that each metabolite causes specific alterations in gene expression that individually contribute to endothelial dysfunction induced by HHcy. Defining metabolite-specific alterations in gene expression in endothelial cells will help to identify molecular pathways involved in the pathology of HHcy and to uncover novel pharmacologic targets for preventing or treating cardiovascular and neurological disorders of HHcy.

An analysis of genes and biological pathways responding to specific Hcy metabolites has not been previously reported. To determine whether changes in gene expression are metabolite-specific and to identify biological pathways altered by HHcy, we studied how treatments with Hcy-thiolactone, *N*-Hcy-protein, and Hcy affect gene expression in human umbilical vein endothelial cells (HUVECs), a frequently used model of vascular cells.

## Materials and methods

### Cell culture and treatments

HUVECs, purchased from Lonza, were seeded into 75-cm^2^ flasks coated with 1 % gelatin and cultured in EGM2 medium (Lonza) at 37 °C/5 % CO_2_, supplemented with 2 % fetal bovine serum (FBS; Sigma–Aldrich), bovine endothelial cell growth factor, and antibiotics: 50 μg/mL penicillin and 100 μg/mL streptomycin (Sigma–Aldrich). Cells between 4 and 10 passage were used in the experiments. After reaching 80–90 % confluence, the cells were treated for 24 h with 0, 10, or 1000 μM D, L-Hcy, or L-Hcy-thiolactone HCl (Sigma–Aldrich), 10 or 40 μM *N*-Hcy-protein in the EGM2/FBS medium without antibiotics.

### *N*-Hcy-protein preparation

*N*-Hcy-protein was prepared by the modification of FBS with Hcy-thiolactone (Jakubowski 1999). Such preparation mimics *N*-Hcy-protein that was present in the plasma of cystathionine β-synthase (CBS) and methylenetetrahydrofolate reductase (MTHFR)-deficient patients (Jakubowski et al. 2008). FBS (0.2 mL, 50 mg protein/ml) was incubated with 5 or 25 mM L-Hcy-thiolactone∙HCl (Sigma–Aldrich) in 0.1 M phosphate buffer pH 7.4, 1 mM EDTA (buffer A) at 37 °C for 16 h. Hcy and unreacted Hcy-thiolactone were removed by four cycles of dilution with buffer A containing 2 mM dithiothreitol (DTT) and ultrafiltration using a 10-kDa cutoff Millipore device (10,000x*g*, 4 °C, 30 min). DTT was then removed by three cycles of dilution with 0.2 mL buffer A and ultrafiltration, and the *N*-Hcy-protein preparation was diluted to 50 mg protein/mL with buffer A. The thiol content in *N*-Hcy-protein was quantified according to Ellman (1959). These *N*-Hcy-protein preparations were added to cell culture media to constitute 2 % of the medium volume, resulting in *N*-Hcy-protein concentrations of 10.1 and 41.1 μM, respectively, similar to the range of *N*-Hcy-protein concentrations observed in disorders of Hcy/folate metabolism in humans (Jakubowski et al. 2008) and mice (Jakubowski et al. 2009).

### Protein thiols quantification

The thiol content in *N*-Hcy-protein was quantified colorimetrically at 412 nm by using 5,5′-dithiobis-2-nitrobenzoic acid (Sigma–Aldrich) and a molar extinction coefficient 13,600 M^−1^ cm^−1^ for 5-nitrothiobenzoate according to Ellman (1959). After modification of serum protein with 0.2 mM Hcy-thiolactone, the protein thiol content increased to 1.18 from a reference value of 1.0 in untreated serum protein. Increasing Hcy-thiolactone concentration to 1, 5, and 25 increased the thiol content in *N*-Hcy-protein to 1.72, 2.34, and 3.74 thiol groups, respectively. Since albumin with its single Cys34 free thiol group is the major contributor to serum protein thiol content (Glowacki and Jakubowski 2004), these values indicate that the modification with Hcy-thiolactone afforded 0.18, 0.72, 1.34, and 2.74 mol *N*-linked Hcy per mol serum protein (albumin).

### RNA extraction

Total RNA enriched in small RNA (>200 bp) was isolated from HUVECs treated with Hcy-thiolactone, *N*-Hcy-protein, Hcy, or untreated control cells using miRvana miRNA Isolation Kit (Life Technologies), according to the supplier’s protocol. Total RNA concentration was measured by using Nano Drop UV/Vis spectrophotometer (2000c, Thermo Scientific). Total RNA quality was assessed by capillary electrophoresis with 2100 Agilent Bioanalyzer (Agilent Technologies). Only high-quality total RNA (RIN > 8.0) was used in the experiments.

### cDNA microarray procedures

Total RNA (20 μg) was reverse-transcribed to cDNA using aminoallyl-modified nucleotides. In the next step, cDNA was fluorescently labeled (Superscript Indirect cDNA Labeling System, Invitrogen) according to the manufacturer’s protocol. Control cDNA was labeled with Alexa 555, while cDNA prepared from RNA isolated from Hcy-, HTL-, or *N*-Hcy-protein-treated HUVECs was labeled with Alexa 647. The amount of linked fluorescent dye was measured by Nano Drop UV/Vis spectrophotometer (2000c, Thermo Scientific). Only samples with at least 100 pmol of the dye linked were used in the assay. Each pair of cDNA samples (from control and treated cells) was mixed 1:1 with hybridization buffer (500 mM NaH2PO4, 2 % SDS, 2 mM EDTA, 2 × SSC), denatured (95 °C, 3 min), kept at 42 °C, and transferred onto OneArray Human Microarrays V4 (Phalanx Biotech Group). Directly before use, microarrays were cross-linked using DNA Crosslinker CL E508 G (Uvitec, 0,18 J), baked at 60 °C for 10 min in SciGene hybridization oven (Oxford Gene Technology), washed with 100 % ethanol (15-s hold, 20-s shake), and deionized with water. Pre-hybridization was carried out in a pre-warmed (50 °C) buffer containing 250 mM ethanolamine, 0.1 % SDS, and 200 mM Na_2_CO_3_ (1 h, RT). The slides were then washed with 80 % ethanol/10 mM Na_2_CO_3_ and water, centrifuged, and incubated with the labelled cDNA introduced under the coverslips (24 × 60 mm, Menzel Glaser) in a chambered box in the hybridization oven (40 °C, 16–18 h). Following hybridization, the slides were washed three times using a microarray ArrayIt wash station [(1) 1 × SSPE/0.03 % SDS, 2 min, 42 °C; (2) 1 × SSPE, 2 min, RT; (3) 0.1 × SSPE, RT], dried by centrifugation, and scanned with Axon 4200AL microarray scanner (Molecular Devices). Hybridizations were carried out in duplicate.

### Microarray data analysis

Microarray data analysis was carried out as previously described (Schmidt et al. 2011). Microarray images were processed using GenePix Pro version 6.1 software (molecular devices). Raw data (gpr files) were submitted to a normalization procedure using R Bioconductor software version 2.13.1 (Smyth and Speed 2003) and limma package (Smyth and Speed 2003). Within-array normalization was carried out using Loess method, while between-array normalization was carried using Aquantile method. To quantify changes in gene expression, the normalized microarray data were fitted to linear models (Smyth 2004). The final *P* values were adjusted using the FDR correction (Benjamini and Hochberg 1995). Adjusted *P* value <0.05 was considered significant.

### Real-time qPCR

cDNA synthesis was carried out using 1 µg of total RNA and Riveraid H Minus First Strand Synthesis cDNA Kit (Thermo Scientific). Quantitative real-time PCR analysis was carried out in Mx 3005P qPCR System (Agilent Technologies) using DyNAmo HS SYBR Green qPCR Kit (Finnzymes) according to the manufacturer’s protocol. Primers were purchased from GenoMed S.A. (Warsaw, Poland); the sequences are shown in Table 1. Relative expression was calculated using the –ΔΔC_*t*_ method using the SYBR Green fluorescence. A reference gene was HPRT. Each reaction was carried out in duplicate (technical replication) for three separate HUVEC cultures (biological replications).

**Table 1:** List of primers used for RT-qPCR

Gene name	Accession number	Forward primer	Reverse primer
*CBS*	875	GCCAGGTTCGACTCCCCGGA	GCTGGCGTTGCGGTACTGGT
*CD9*	928	CTTCTGGCTTGCCGGGATTGCT	GCGCCGGCTCCGATCAGAAT
*ESM1*	11,082	GCGGTGGACTGCCCTCAACA	CCAGATGCCATGTCATGCTCTTTGC
*OS9*	10,956	GCTGGCATGGAGCGGGAACT	TCTGTGCTCAGGATCCTCCTCTGT
*MTR*	4548	CTGCCAATGCCAAGGCAGCCT	GTCCGGAAAGAGTCCGCCCAC
*MTRR*	4552	CTGGCGCAAGGTTGGTGGAAGT	TAACAGTGAAACCACGCCGCGC
*ANXA8*	653,145	TGGGAAAGGTGCCCCCGAGG	GCCTGCTCGTTGGTCCCGAT
*APOL1*	8542	GGCTGTGCTGTGTCCCTAATGGG	CACAGGCACCATTCTGCAACGC
*APOA5*	116,519	GCCTCCCTCCACCTGTCTTCTCAGA	AGTCCCAGAAGCCTTTCCGTGC
*MARCH1*	55,016	AGAAGTGTGCATAACCATGAGTGGG	TCTGCCTGTCCCTTGCGTCA
*VEGFA*	7422	GGTGCCCGCTGCTGTCTAATG	TCAAGCTGCCTCGCCTTGCAAC
*MMP19*	4327	GCACGGACAGCCTCTGGAGC	ACAGGTAGTCCACGGGCGCC
*NTN4*	59,277	CGGAGGAGGACGCCCAGGAG	GGAGCCCCGGGACCATAGCC
*DDX28*	55,794	GTCCGCAGTCCCGACGAACC	GTCGAACCAGCACCGGCCTC
*SCUBE1*	80,274	CGGGCTTGGTCTGCCTGGTG	CAGGCTGGTACGTGCCCACG
*ROBLD3*	28,956	CGCCTACGACCGGAACGGGA	ACCAAAGCCTGGGCCATGCAG
*SLC44A3*	126,969	GGGCGCCGAGTACCTGGTTTC	CCAGCCACCACCGAGTAGCC
*ST8SIA2*	8128	ATCGTGGGCAACTCGGGGGT	AAGGCCCGCTGGATGACCGA
*NCEH1*	57,552	GAAGAGCCACTGAAACGCAGCG	GACCAGGACGCACTTGCACTTG
*HPRT*	3251	CTGAGGATTTGGAAAGGGTG	AATCCAGCAGGTCAGCAAAG

### Pathway and network analysis

Bioinformatic analysis to discover biological pathways and human diseases linked to genes responding to Hcy-thiolactone, *N*-Hcy-protein, and Hcy was carried out using the PANTHER classification system (http://www.pantherdb.org/) and DAVID Knowledgebase and functional annotation tools (http://david.niaid.nih.gov). The networks containing those genes were identified using the Ingenuity Pathway Analysis software (IPA, Ingenuity Systems, Mountain View, CA). The data sets containing differentially expressed mRNAs identified in microarray and RT-qPCR experiments were uploaded into the IPA Knowledge database, which mapped gene/gene products to global molecular networks to identify genes that are known to interact with other gene products in the database.

### Statistical analysis

RT-qPCR results are expressed as a mean ± SD. One-way analysis of variance was used to determine the significance of RT-qPCR experiments (GraphPad Prism Software). Fisher’s exact test was used to determine *P* values in the pathway and network analyses. In all cases, *P* values <0.05 were considered significant.

## Results

### Identification of genes affected by Hcy and its metabolites in human endothelial cells

**Fig. 1:**
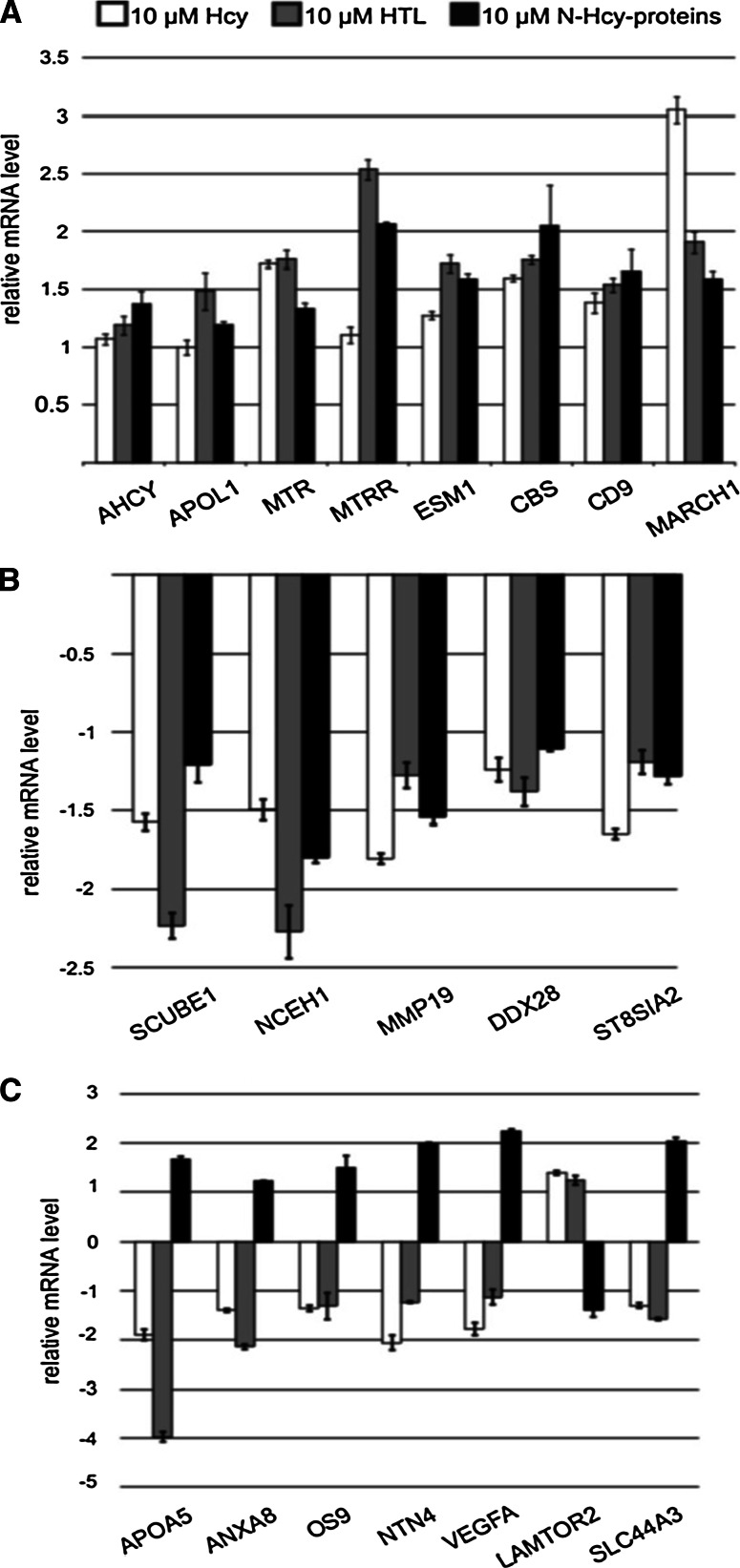
Changes in gene expression induced by Hcy-thiolactone, *N*-Hcy-protein, and Hcy in human endothelial cells. mRNA levels were quantified by RT-qPCR, and values relative to untreated controls are shown. All changes are statistically significant (*P* < 0.05)

The Phalanx Biotech OneArray Human Microarrays version 4, containing 2,918,760 human genome probes and 1088 experimental control probes, was used to interrogate 31,741 human genes in HUVECs treated with low and high concentrations of Hcy-thiolactone, *N*-Hcy-protein, and Hcy. Of these genes, 110 were significantly altered in response to Hcy-thiolactone treatment (Table 2), 30 mRNAs were altered in response to *N*-Hcy-protein (Table 3), and 14 mRNAs were altered in response to Hcy (Table 4). In general, the magnitude of the expression change increased at high metabolite concentrations. Additionally, 3, 17, and 16 genes whose expression was altered by Hcy-thiolactone, *N*-Hcy-protein, or Hcy, respectively were identified by RT-qPCR (Fig. 1).

**Table 2:** List of Hcy-thiolactone-responsive genes and their expression levels

Gene name	Accession number	Protein name	Rel. expression, fold	
			10 μM Hcy-thiolactone	1000 μM Hcy-thiolactone
*ADSSL1*	122,622	Adenylosuccinate synthase like 1	2.08	3.13
*AHCY*	191	Adenosylhomocysteinase	3.63	4.29
*ALDH1L1*	10,840	Aldehyde dehydrogenase 1 family, member L1	2.98	7.92
*AMOTL2*	51,421	Angiomotin like 2	3.24	9.48
*ANGPT2*	285	Angiopoietin 2	2.64	2.16
*ANGPT4*	51,378	Angiopoietin 4	2.55	3.12
*ANGPTL3*	27,329	Angiopoietin-like 3	−2.16	−2.71
*ANTXR2*	118,429	Anthrax toxin receptor 2	1.59	4.8
*ANXA8*	653,145	Annexin A8	−2.21	−4.66
*APOA1B*	128,240	Apolipoprotein A-I binding protein	3.73	2.86
*APOA4*	337	Apolipoprotein A-IV	2.68	6.23
*APOA4*	107,690	Apolipoprotein A-IV	2.68	2.68
*APOA5*	116,519	Apolipoprotein A-V	−2.59	−7.07
*APOB*	338	Apolipoprotein B	2.4	5.29
*APOL1*	8542	Apolipoprotein L1	3.79	5.86
*APOL5*	80,831	Apolipoprotein L5	2.3	4.93
*APOL6*	80,830	Apolipoprotein L6	2.58	3.32
*APOLD1*	81,575	Apolipoprotein L domain containing 1	−1.13	−5.56
*APOO*	79,135	Apolipoprotein O	2.27	4.82
*BRD8*	10,902	Bromodomain containing 8	−2.79	−4.78
*BTN3A2*	11,118	Butyrophilin, subfamily 3, member A2	1.61	2.9
*C1QTNF4*	114,900	C1q and tumor necrosis factor related protein 4	−3.18	−3.52
*CBS*	875	Cystathionine β-synthase	4.73	10.6
*CBX2*	12,416	Chromobox 2	−5.97	−7.06
*CCDC129*	223,075	Coiled-coil domain containing 129	−2.51	−2.14
*CD209*	30,835	CD209 molecule	−2.1	−4.28
*CD9*	928	CD9 molecule	3.55	11.75
*CH25H*	9023	Cholesterol 25-hydroxylase	1.32	2.53
*CRSP7*	9441	Mediator complex subunit 26	1.68	4.4
*CYBASC3*	220,002	Cytochrome b561 family, member A3	2.01	3.63
*CYP2S1*	29,785	Cytochrome P450, family 2, subfamily S, polypeptide 1	2.74	6.66
*DDIT4L*	115,265	DNA-damage-inducible transcript 4-like	1.39	3.46
*DOT1L*	84,444	DOT1-like, histone H3 methyltransferase	−2.52	−2.19
*EHMT1*	79,813	Euchromatic histone-lysine *N*-methyltransferase 1	2.02	3.69
*EHMT2*	10,919	Euchromatic histone-lysine *N*-methyltransferase 2	1.69	2.46
*ELP2*	55,250	Elongator acetyltransferase complex subunit 2	2.09	2.41
*EP300*	2033	E1A binding protein p300	2.06	2.79
*EPC1*	80,314	Enhancer of polycomb homolog 1	1.77	2.02
*ESM1*	11,082	Endothelial cell-specific molecule 1	2.25	6.59
*EZH2*	2146	Enhancer of zeste homolog 2	2.31	5.36
*FABP3*	2170	FABP3 fatty acid binding protein 3, muscle and heart	3.73	4.97
*FGB*	2244	Fibrinogen beta chain	−2.36	−2.45
*H2AFY*	9555	H2A histone family, member Y	1.71	2.46
*HDLBP*	3069	High-density lipoprotein binding protein	−1.29	−4.57
*HERPUD2*	64,224	Homocysteine-responsive endoplasmic reticulum-resident ubiquitin-like domain member 2	4.61	5.79
*HIST1H1B*	3009	Histone cluster 1, H1b	−7.7	−9.38
*HIST1H2AJ*	8330	Histone cluster 1, H2ak	−2.73	−4.12
*HIST1H2BK*	85,236	Histone cluster 1, H2bk	2.26	2.46
*HPSE2*	60,495	Heparanase 2	2.73	6.64
*IBRDC2*	255,488	E3 Ubiquitin-protein ligase IBRDC2	−4.24	−3.94
*ICAM1*	3383	Intercellular adhesion molecule 1	2.56	17.3
*IFI30*	10,437	Interferon gamma-inducible protein 30	1.11	3.7
*IL20RA*	53,832	Interleukin 20 receptor, Ralpha	−2.18	−2.14
*IRF2*	3660	Interferon regulatory factor 2	−2.3	−3.75
*JARID2*	3720	jumonji, AT rich interactive domain 2	3.57	2.7
*JMJD2B*	23,030	Lysine (K)-specific demethylase 4B	−1.87	−2.98
*KRT27*	342,574	Keratin 27	−2.55	−6.45
*LAMTOR2*	28,956	Late endosomal/lysosomal adaptor, MAPK and MTOR activator 2	2.24	3.65
*LMX1A*	4009	LIM Homeobox transcription factor 1, alpha	−2.14	−6.27
*LPL*	4023	Lipoprotein lipase	1.61	4.03
*LRP8*	7804	Low-density lipoprotein receptor-related protein 8, apolipoprotein e receptor	3.49	5.02
*LRRIQ1*	84,125	Leucine-rich repeats and IQ motif containing 1	−5.97	−8.91
*MAT1A*	4143	Methionine adenosyltransferase I	4.46	22.06
*MARCH1*	55,016	Membrane-associated ring finger (C3HC4) 1, E3 ubiquitin-protein ligase	4.14	5.46
*MB*	4151	Myoglobin	4.22	18.66
*MBD3*	53,615	Methyl-CpG-binding domain protein 3	1.54	2.39
*MMP19*	4327	Matrix metallopeptidase 19	−2.45	−12.75
*MMRN1*	22,915	Multimerin 1	−2.68	−6.25
*MSR1*	4481	Macrophage scavenger receptor 1	3.43	2.15
*MTHFD2*	10,797	Methylenetetrahydrofolate dehydrogenase 2, methenyltetrahydrofolate cyclohydrolase	2.2	4.58
*MTR*	4548	5-Methyltetrahydrofolate-homocysteine methyltransferase	2.84	15.42
*MTRR*	4552	5-Methyltetrahydrofolate-homocysteine methyltransferase reductase	2.31	6.27
*NCEH1*	57,552	Neutral cholesterol ester hydrolase 1	2.64	9.95
*NSD1*	64,324	Nuclear receptor binding SET domain protein 1	2.52	3.16
*NT5DC4*	284,958	5′-Nucleotidase domain containing 4	1.26	3.9
*NTN4*	59,277	Netrin 4	−2.87	−12.24
*PAPPA2*	60,676	Pappalysin 2	1.58	2.99
*PCDHB7*	56,129	Protocadherin beta 7	−2.33	−5.52
*PFK*	5211L	Phosphofructokinase, liver	−2.48	−2.72
*PHACTR*	465,979	Phosphatase and actin regulator 4	−2.47	−8.93
*PIGL*	9487	Phosphatidylinositol glycan Anchor biosynthesis, class L	−2.73	−2.31
*PLG*	5340	Plasminogen	2.76	6.76
*PPAP2B*	8613	Phosphatidic acid phosphatase type 2B	3.73	8.05
*PPAP2C*	607,126	Phosphatidic acid phosphatase type 2C	2.41	6.29
*PTPN6*	5777	Protein tyrosine phosphatase, non-receptor type 6	−3.13	−12.12
*PTPRB*	5787	Protein tyrosine phosphatase, receptor type, B	−4.07	−16.67
*SCARF1*	8578	Scavenger receptor class F, member 1	1.51	3.65
*SCL44A3*	126,969	Solute carrier family 44, member 3	−1.27	−5.32
*SCUBE1*	80,274	Signal peptide, CUB domain, EGF-like 1	−2.19	−12.37
*SERPINA9*	327,657	Serpin peptidase inhibitor, clade A (alpha-1 antiproteinase, antitrypsin), member 9	3.67	12.73
*SETD2*	29,072	Histone-lysine *N*-methyltransferase SETD2 (EC 2.1.1.43)	17.93	6.08
*SETD7*	80,854	Lysine methyltransferase 7	1.07	3.9
*SHMT1*	6470	Serine hydroxymethyltransferase 1	−1.13	−2.43
*SLC22A9*	114,571	Solute carrier family 22 (organic anion transporter), member 9	2.45	4.42
*SLC2A12*	154,091	Solute carrier family 2 (facilitated glucose transporter), member 12	2.19	2.45
*SMOC2*	64,094	SPARC related modular calcium binding 2	1.59	3.02
*SMYD3*	64,754	SET and MYND domain containing 3	−2.75	−11.25
*SUV420H2*	84,787	Suppressor of variegation 4–20 homolog 2	−1.89	−3.33
*THAP1*	55,145	THAP domain containing, apoptosis associated protein 1	2.72	9.99
*TLE2*	7089	Transducin-like enhancer of split 2	1.07	3.17
*TNFRSF12A*	51,330	Tumor necrosis factor receptor superfamily, member 12A	2.07	4.19
*TNNI3*	7137	Troponin I type 3 (cardiac)	2.22	2.66
*TNNT2*	7139	Troponin T type 2 (cardiac)	3.27	9.67
*UBE2G1*	7326	Ubiquitin-conjugating enzyme E2G 1	1.79	2.36
*USP11*	8237	Ubiquitin-specific peptidase 11	−4.13	−6.17
*VAV3*	10,451	Vav 3 guanine nucleotide exchange factor	4.58	13.84
*VCAM1*	7412	Vascular cell adhesion molecule 1	2.17	4.31
*VEGFA*	7422	Vascular endothelial growth factor A	−4.37	−7.37
*VWF*	7450	Von Willebrandt factor	3.67	6.44
*ZNF135*	1,357,694	Zinc finger protein 135	−1.21	−3.39

**Table 3:** List of *N*-Hcy-protein-responsive genes and their expression levels

Gene name	Accession number	Protein name	Rel. expression, fold	
			10 μM *N*-Hcy-protein	40 μM *N*-Hcy-protein
*BCNP1*	199,786	Family with sequence similarity 129, member C	−1.1	−6.01
*CGI*-*12*	51,001	MTERF domain containing 1	−1.14	−8.97
*CHD5*	1003	Cadherin 5, type 2 (vascular endothelium)	1.16	3.95
*CORIN*	10,699	Corin, serine peptidase	−6.36	−21.04
*DDX28*	55,794	DEAD (Asp-Glu-Ala-Asp) box polypeptide 28	−6.11	−22.04
*DNAJB7*	150,353	DnaJ (Hsp40) homolog, subfamily B, member 7	2.47	10.41
*DOCK1*	1793	Dedicator of cytokinesis 1	−8.02	−15.11
*DPEP2*	64,174	Dipeptidase 2	−1.39	−22.66
*EDF1*	8721	Endothelial differentiation-related factor 1	−1.98	−2.25
*EEF1A1*	1915	Eukaryotic translation elongation factor 1 alpha 1	−2.5	−46.91
*EIF3S6IP*	51,386	Eukaryotic translation initiation factor 3, subunit 6 interacting protein	−1.02	−8.88
*EPHX1*	2052	Epoxide hydrolase 1, microsomal	4.71	9.53
*ESM1*	11,082	Endothelial cell-specific molecule 1	19.6	10.55
*KRT25C*	147,183	Keratin 25C	−3.86	−4.06
*LOC40139*	4013	Hypothetical gene supported by BC063892	−1.1	−4.54
*LRRIQ1*	84,125	Leucine-rich repeats and IQ motif containing 1	−1.15	−30.41
*MELL1*	27,390	Mel transforming oncogene-like 1	−1.75	−48.02
*MPO*	4353	Myeloperoxidase	−6.36	−4.66
*MRPS5*	64,969	Mitochondrial ribosomal protein S5	2.56	12.55
*MYL6*	4637	Myosin, light-chain 6	1.68	7
*MYL12B*	103,910	Myosin, light-chain 12B, regulatory	1.79	48.46
*OS9*	10,956	Osteosarcoma amplified 9, endoplasmic reticulum lectin	2.41	21.88
*PHACTR4*	65,979	Phosphatase and actin regulator 4	−1.21	−4.69
*PLB1*	151,056	Phospholipase B1	1.14	2.26
*PRKAB2*	5565	Protein kinase, AMP-activated, beta 2 non-catalytic subunit	−3.13	−9.81
*RP11*-*30M20.2*	26,121	PRP31 pre-mRNA processing factor 31 homolog (S. cerevisiae)	2.56	4.12
*SLC25A18*	83,733	Solute carrier family 25 (glutamate carrier), member 18	4.99	8.67
*SPRY3*	10,251	Sprouty homolog 3 (Drosophila)	−1.49	−10.74
*SULT1E1*	6783	Sulfotransferase family 1E, estrogen-preferring, member 1	−1.7	−9.26
*ZNF227*	1,357,694	Zinc finger protein 227	−1.39	−29.87

**Table 4:** List of Hcy-responsive—genes and their expression levels

Gene name	Accession number	Protein name	Rel. expression, fold	
			10 μM Hcy	1 mM Hcy
*CD209*	30,835	CD209 molecule	−1.1	−3.68
*DNAJC2*	129,450	DnaJ homolog subfamily C member 28	1.18	3.99
*IFI30*	10,437	Interferon gamma-inducible protein 30	1.83	3.71
*IGIP*	492,311	IgA-inducing protein homolog (Bos taurus)	−1.93	−3.09
*KRT27*	342,574	Keratin 27	−1.18	−6.55
*LAMTOR2*	28,956	Late endosomal/lysosomal adaptor, MAPK and MTOR activator 2	1.21	4.9
*LMX1A*	4009	LIM homeobox transcription factor 1, alpha	2.58	2.69
*LRRIQ1*	84,125	Leucine-rich repeats and IQ motif containing 1	−2.22	−116.1
*MARCH1*	55,016	Membrane-associated ring finger (C3HC4) 1, E3 ubiquitin-protein ligase	1.28	3.72
*PCDHB7*	56,129	Protocadherin beta 7	−1.35	−7.17
*PHACTR4*	65,979	Phosphatase and actin regulator 4	−1.31	−7.61
*SLC22A9*	114,571	Solute carrier family 22 (organic anion transporter), member 9	1.48	7.14
*SLC44A3*	126,969	Solute carrier family 44, member 3	−2.68	−12.47
*ST8SIA2*	8128	ST8 alpha-*N*-acetyl-neuraminide alpha-2,8-sialyltransferase 2	−1.42	−10.56

### Limited overlap between groups of genes regulated by *N*-Hcy-protein, Hcy-thiolactone, and Hcy

**Fig. 2:**
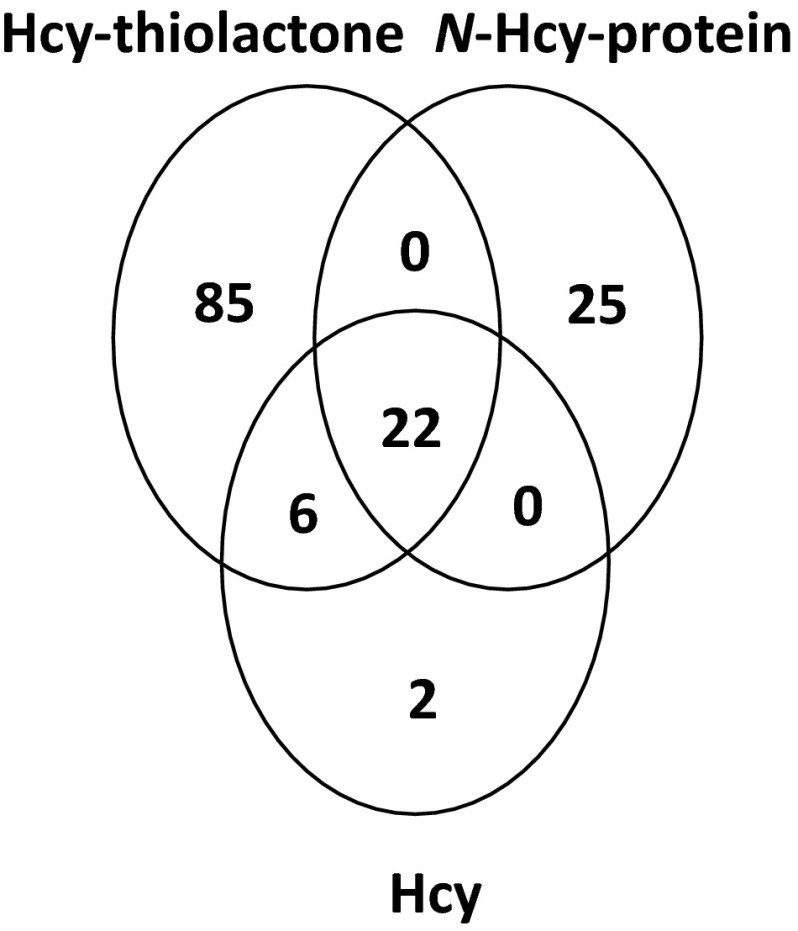
Wenn diagram illustrating limited overlap between genes affected by Hcy-thiolactone, *N*-Hcy-protein, and Hcy

Only a minority of Hcy-thiolactone-affected genes was also affected by Hcy (28 out of 113) or by *N*-Hcy-protein (22 out of 113). Likewise, only some of *N*-Hcy-protein-regulated genes were also regulated by Hcy-thiolactone (22 out of 47) or Hcy (22 out of 47) (Fig. 2). However, the majority of genes responding to Hcy also responded to Hcy-thiolactone (28 out of 30) and *N*-Hcy-protein (22 out of 30). There were genes that responded only to one metabolite: Hcy-thiolactone (87 genes), *N*-Hcy-protein (25 genes), or Hcy (2 genes). Twenty-two genes responded to all three metabolites (Table 5).

**Table 5:** List of genes whose expression was altered by all three metabolites

Gene name	Accession number	Protein name	Change in expression induced by		
			*N*-Hcy-protein	Hcy-thiolactone	Hcy
*AHCY*	191	Adenosylhomocysteinase	↑	↑	↑
*ANXA8*	653,145	Annexin A8	↑	↓	↓
*APOA5*	116,519	Apolipoprotein A-V	↑	↓	↓
*APOL1*	8542	Apolipoprotein L1	↑	↑	
*CBS*	875	Cystathionine β-synthase	↑	↑	↑
*CD9*	928	CD9 molecule	↑	↑	↑
*DDX28*	55,794	DEAD (Asp-Glu-Ala-Asp) box polypeptide 28	↓	↓	↓
*ESM1*	11,082	Endothelial cell-specific molecule 1	↑	↑	↑
*LAMTOR2*	28,956	Late endosomal/lysosomal adaptor, MAPK and MTOR activator 2	↓	↑	↑
*LRRIQ1*	84,125	Leucine-rich repeats and IQ motif containing 1	↓	↓	↓
*MMP19*	4327	Matrix metallopeptidase 19	↓	↓	↓
*MTR*	4548	5-Methyltetrahydrofolate-homocysteine methyltransferase	↑	↑	↑
*MTRR*	4552	5-Methyltetrahydrofolate-homocysteine methyltransferase reductase	↑	↑	↑
*NCEH1*	57,552	Neutral cholesterol ester hydrolase 1	↓	↓	↓
*NTN4*	59,277	Netrin 4	↑	↓	↓
*OS9*	10,956	Osteosarcoma amplified 9, endoplasmic reticulum lectin	↑	↓	↓
*PHACTR4*	65,979	Phosphatase and actin regulator 4	↓	↓	↓
*SCUBE1*	80,274	Signal peptide, CUB domain, EGF-like 1	↓	↓	↓
*SLC44A3*	126,969	Solute carrier family 44, member 3	↑	↓	↓
*ST8SIA2*	8128	ST8 Alpha-*N*-acetyl-neuraminide alpha-2,8-sialyltransferase 2	↓	↓	↓
*VEGFA*	7422	Vascular endothelial growth factor A	↑	↓	↓

Up (↑) and down (↓) arrows indicate up-regulated and down-regulated gene expression

Hcy-thiolactone, *N*-Hcy-protein, and Hcy regulated 22 common genes: seven were down-regulated (*DDX28*, *LRRIQ1*, *MMP19*, *NCEH1*, *PHACTR4*, *SCUBE1*, *ST8SIA2*) and eight were up-regulated (*AHCY*, *APOL1*, *MTR*, *MTRR*, *ESM1*, *CBS*, *CD9*, *MARCH1*). Seven other genes were regulated in the opposite direction by *N*-Hcy-protein, compared to Hcy and Hcy-thiolactone: *APOA5*, *ANXA8*, *NTN4*, *OS9*, *SLC44A3*, and *VEGFA* were up-regulated by *N*-Hcy-protein, but down-regulated by Hcy and Hcy-thiolactone, while *LAMTOR2* was down-regulated by *N*-Hcy-protein and up-regulated by Hcy and Hcy-thiolactone. Hcy-thiolactone and Hcy down-regulated four genes (*CD209*, *KRT27*, *LMX1A*, *PCDHB7*) and up-regulated two (*IFI30*, *SLC22A9*).

### Genes affected by Hcy-thiolactone

Of the 113 genes affected by Hcy-thiolactone treatment, seventy-one were up-regulated and forty-two were down-regulated (Table 2). Significant changes in gene expression occurred in endothelial cells treated with 10 μM Hcy-thiolactone and were enhanced at 1000 μM Hcy-thiolactone. The up-regulation varied from 2.02-fold for the regulator of angiogenesis *EPC1* gene to 22.06-fold for the *S*-adenosylmethionine synthase *MAT1A* gene. The down-regulation was from −2.14-fold for the interleukin-20 receptor subunit alpha *IL20RA* gene to −16.67-fold for the protein tyrosine phosphatase beta *PTPRB* gene.

Genes up-regulated by Hcy-thiolactone include those involved in chromatin modification/assembly, histone methylation/transcription (*H2AFY*, *HIST1H2BK*, *CRSP7*, SETD7, *EHMT1*, *EHMT2*, *EPC1*, *NSD1*, *SETD2*, *EZH2*, *EP300*, *JARID2*, *ELP2*, *MBD3*), folate and one-carbon metabolism (*MAT1A*, *AHCY*, *MTR*, *MTRR*, *CBS*, *ALDH1L1*, *MTHFD2*), lipid transport (*APOA4*, *APOA1B*, *APOL1*, *APOL5*, *APOL6*, *APOB*, *APOO*, *FABP3*, *MSR1*), lipid metabolism (*LPL*, *CH25H*), cell adhesion (*ICAM1*, *VCAM1*, *CD9*, *VWF*, *SCARF1*, *SMOC2*, *ANTXR2*), angiogenesis (*VAV3*, *ESM1*, *ANGPT4, SERPINEF1*), proteolysis (*SERPINA9*, *PAPPA2*, *UBE2G1*), endothelial cell cycle (*THAP1*), cytoskeleton function (*TNNT2*, *TNNI3*), Wnt signaling (*AMOTL2*, *PPAP2B*, *TLE2*), mTOR signaling (*LAMTOR2*, *DDIT4L*), endocytotic signaling (*LRP8*), apoptotic signalling (*TNFRSF12A*), phospholipid metabolism (*PPAP2B*, *PPAP2C*), blood clotting (*PLG*), oxygen transport (*MB*), detoxification (*CYP2S1*), extracellular matrix organization (*HPSE2*), purine biosynthesis (*ADSSL1*), nucleotide metabolism (*NT5DC4*), endoplasmic reticulum stress (*HERPUD2*), organic anion transport (*SLC22A9*), glucose transport (*SLC2A12*), lysosome function (*CYBASC3*), and immunity (antigen processing—*IFI30*; protein ubiquitination—*MARCH1*; inhibition of IFNG release from T cells—*BTN3A2*).

Genes down-regulated by Hcy-thiolactone include those involved in chromatin modification/transcription regulation (H2AK119 ubiquitination *CBX2*; methylation—*DOT1L*, *SUV420H2*, *SMYD3*; demethylation—*JMJD2B*), nucleosome assembly (*HIST1H2AJ*, *HIST1H1B*, *BRD8*), one-carbon metabolism (*SHMT1*), lipid metabolism/transport (*NCEH1*, *ANGPTL3*, *APOA5*, *HDLBP*), angiogenesis (*ANGPT2*, *APOLD1*, *VEGFA*, *MMP19*, *PTPRB*), blood coagulation (*FGB*, *ANXA8, IRF2*), cell adhesion (*PCDHB7*, *MMRN1*), immunity/cell adhesion (*CD209*), energy metabolism/glycolysis (*PFK*), cytokine signaling (*IL20RA*, *C1QTNF4*), signaling (*PHACTR4*, *PTPN6*), glycolipid biosynthesis (*PIGL*), cell morphology (*ZNF135*), protein ubiquitination (*IBRDC2*), protein deubiquitination (*USP11*), transmembrane transport (choline transporter—*SLC44A3*), cytoskeleton assembly (*KRT27*), extracellular matrix organization (*NTN4*), endoplasmic reticulum quality control (*OS9*), N-glycan processing (*ST8SIA2*), and unknown function (*CCDC129*, *LRRIQ1*, *SCUBE1*).

It should be noted that in HUVEC cultures, Hcy-thiolactone turns over with a half-life of 3.5 h (Jakubowski et al. 2000). Thus, when 10 μM Hcy-thiolactone is added to cell cultures, its concentration drops by 2-fold every 3.5 h and reaches 70 nM at the end of a 24-h treatment. This range of Hcy-thiolactone concentrations corresponds to the values observed in human plasma from CBS-deficient patients and in mouse models of HHcy (Chwatko et al. 2007).

### Genes affected by *N*-Hcy-protein

Treatment of HUVECs with *N*-Hcy-protein caused changes in the expression of forty-seven genes (Table 3). Significant changes in mRNA levels were observed when the cells were treated with 10.1 μM *N*-Hcy-protein and were enhanced at 40.1 μM *N*-Hcy-protein. Eleven mRNAs were up-regulated and nineteen were down-regulated by *N*-Hcy-protein. The up-regulation varied from 2.3-fold for the phospholipase B1 *PLB1* gene to 48.5-fold for the myosin light-chain 12B *MYL12B* gene. The down-regulation was from −2.3-fold for the endothelial differentiation-related factor 1 *EDF1* gene to −46.9-fold for the eukaryotic translation elongation factor 1 alpha 1 *EEF1A1* gene.

Genes up-regulated by *N*-Hcy-protein include those involved in the regulation of cell morphology (*MYL12B*, *MYL6*), endoplasmic reticulum quality control (*OS9*), protein biosynthesis (*MRPS5*), angiogenesis (endothelial cell-specific molecule 1—*ESM1*), protein folding (*DNAJB7*), mRNA splicing (*PRP31*), transcription regulation (ATP-dependent helicase—*CHD5*), detoxification (epoxide hydrolase 1—*EPHX1*), membrane transport (mitochondrial glutamate carrier 2—*SLC25A18*), and lipid metabolism (phospholipase B1—*PLB1*).

Genes down-regulated by *N*-Hcy-protein include those involved in phagocytosis (*DOCK1*), proteolysis (*MELL1*), protein biosynthesis (*EIF3S6IP*, *EEF1A1*, *BCNP1*), peptide hormone processing (*CORIN*), transcription regulation (*ZNF227*, *MTERFD1*, *EDF1*), RNA processing or transport (*DDX28*), signal transduction (*SPRY3*, *PHACTR4*), cytoskeleton assembly (*KRT25C/27*), energy metabolism (*PRKAB2*), leukotriene metabolism (*DPEP2*), host defense system (*MPO*), sulfation (*SULT1E1*), *N*-glycan processing (*ST8SIA2*), and unknown function (*LOC4013*, *LRRIQ1*).

### Genes affected by Hcy

Of the 30 genes whose expression was changed in endothelial cells treated with Hcy, 17 were up-regulated and 13 were down-regulated (Fig. 1; Table 4). Genes up-regulated by Hcy include those involved in transmembrane transport (organic anion transporter—*SLC22A9*), mTOR signaling (*LAMTOR2*), transcription activation (*DNAJC2*), immunity (antigen processing—*IFI30*; protein ubiquitination—*MARCH1*).

Genes down-regulated by Hcy include those involved in transmembrane transport (choline transporter—*SLC44A3*), transcription activation (*LMX1A*), immunity (IgA secretion—*IGIP*, cell adhesion—*CD209*), cell adhesion (*PCDHB7*), neurogenesis/signal transduction (*PHACTR4*), *N*-glycan processing (*ST8SIA2*), cytoskeleton assembly (*KRT27*), endoplasmic reticulum quality control (*OS9*), and unknown function (*LRRIQ1*) (Table 4).

It should be noted that Hcy can be attached to proteins, mostly the conserved Cys34 of albumin, via disulfide bonds and form *S*-Hcy-protein (Hortin et al. 2006; Jakubowski 2000, 2002b; Sengupta et al. 2001). Thus, it is possible that some of the changes in HUVEC gene expression in response to the supplementation with Hcy can be caused by *S*-Hcy-protein.

### Validation of microarray analyses by RT-qPCR

To validate microarray results, we selected 20 genes that showed at least 4-fold change in mRNA level in response to *N*-Hcy-protein, Hcy-thiolactone, or Hcy and examined their expression by RT-qPCR (Fig. 1). For each of the 20 examined genes, RT-qPCR confirmed the microarray mRNA expression results. Genes such as *AHCY*, *APOL1*, *MTR*, *MTRR*, *ESM1*, *CBS*, *CD9*, *LAMTOR2*, and *MARCH1* that were up-regulated by Hcy-thiolactone (Table 2); *OS9* and *DDX28* that were up-regulated by *N*-Hcy-protein (Table 3); *LAMTOR2* and *ST8SIA2* that were up-regulated by Hcy (Table 4) in microarray experiments were also found to be up-regulated in RT-qPCR experiments (Fig. 1a). *SCUBE1*, *NCEH1*, *MMP19*, *DDX28*, and *ST8SIA2* that were down-regulated by Hcy-thiolactone in microarray experiments (Table 2) were also found to be down-regulated in RT-qPCR experiments (Fig. 1b). Some genes that were divergently regulated by each metabolite in microarray experiments (*APOA5*, *ANXA8*, *NTN4*, *VEGFA*, *OS9*, *LAMTOR2*, *SLC44A3*; Tables 2, 3, 4) were also found to be divergently regulated in RT-qPCR experiments (Fig. 1c).

## Bioinformatic analysis

### Biological pathways and diseases

**Fig. 3:**
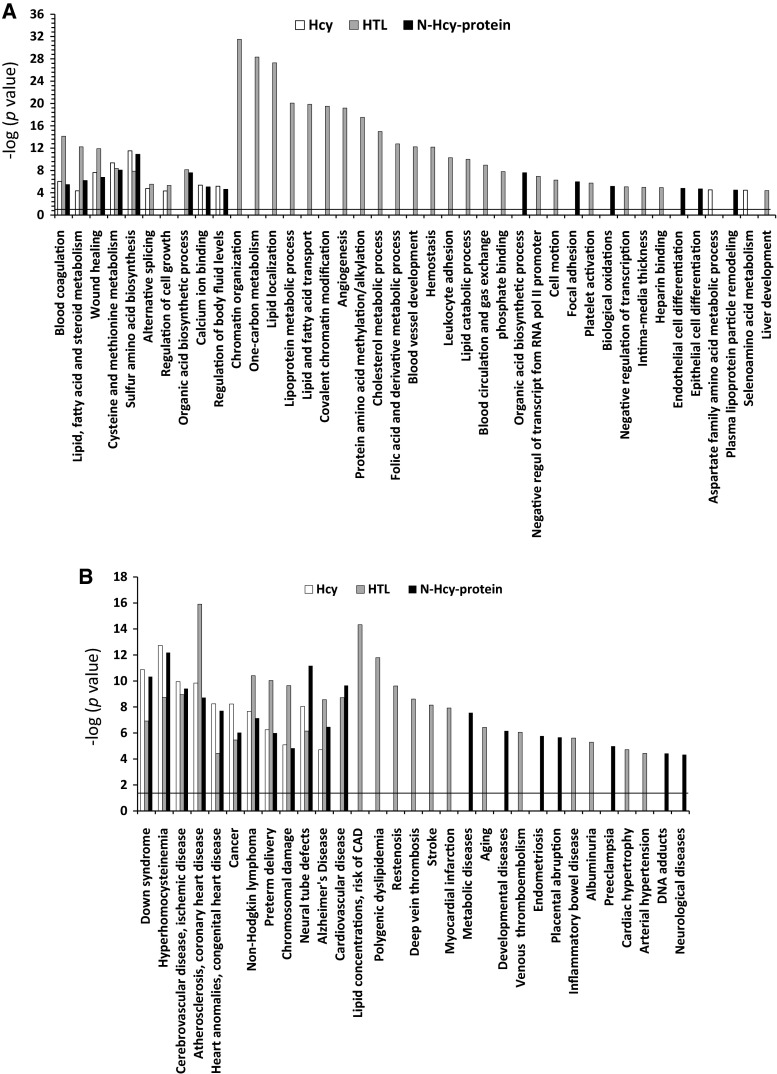
Molecular pathways and disease associated with Hcy-thiolactone, *N*-Hcy-protein, and Hcy identified by DAVID tool. The analysis utilized *P* value 0.05, and Benjamini–Hochberg, Bonferroni, and FDR corrections were applied for minimizing the number of false positives. Panel A, molecular pathways; Panel B, diseases

To identify biological pathways and human diseases linked to genes responding to Hcy-thiolactone, *N*-Hcy-protein, or Hcy, we carried out bioinformatic analysis using the PANTHER classification system and DAVID functional annotation tools. We found that genes affected by Hcy-thiolactone were significantly enriched in thirty molecular pathways, while genes affected by *N*-Hcy-protein or Hcy were enriched in thirteen or eleven molecular pathways, respectively. Only five common pathways were enriched in genes affected by all three metabolites (Fig. 3a). Overall, there was very little overlap between molecular pathway categories enriched in genes affected by Hcy-thiolactone, *N*-Hcy-protein, or Hcy (Fig. 3a).

The majority of Hcy-thiolactone-affected pathway categories were not affected by *N*-Hcy-protein or Hcy. Top five highly significant [−log(*P* value) = 20–31] pathway categories affected only by Hcy-thiolactone were ‘chromatin organization,’ ‘one-carbon metabolism,’ ‘lipid localization,’ ‘lipoprotein metabolic processes,’ and ‘lipid and fatty acid transport’ (Fig. 3a).

Most of the molecular pathway categories that were affected by *N*-Hcy-protein or Hcy were also affected by Hcy-thiolactone. Five common pathways were enriched in genes affected by the three metabolites [−log(*P* value) = 4–11]: ‘blood coagulation’; ‘sulfur amino acid biosynthesis’; ‘cysteine and methionine metabolism’; ‘wound–healing’; and ‘lipid, fatty acid, and steroid metabolism’ [these pathways were also affected by Hcy-thiolactone, −log(*P* value) = 8–14] (Fig. 3a).

Six molecular pathway categories were enriched in genes affected only by *N*-Hcy-protein [−log(*P* value) = 4–7]: ‘organic acid biosynthetic process,’ ‘focal adhesion,’ ‘biological oxidations,’ ‘endothelial cell differentiation,’ ‘epithelial cell differentiation,’ ‘plasma lipoprotein particle remodeling.’ Only two molecular pathways were enriched in genes affected only by Hcy [−log(*P* value) = 4]: ‘aspartate family amino acid metabolic process’ and ‘seleno amino acid metabolism.’

Eleven disease categories were found to be significantly related [−log(*P* value) = 4–16] to all three metabolites Hcy-thiolactone, *N*-Hcy-protein, and Hcy (Fig. 3b): ‘down syndrome’; ‘hyperhomocysteinemia’; ‘cerebrovascular disease, ischemic disease’; ‘atherosclerosis, coronary heart disease’; ‘heart anomalies, congenital heart disease’; ‘cancer’; ‘non-Hodgkin lymphoma’; ‘preterm delivery’; ‘chromosomal damage’; ‘neural tube defects’; and ‘Alzheimer’s disease.’ One disease category ‘cardiovascular disease’ was significantly related to Hcy-thiolactone and *N*-Hcy-protein [−log(*P* value) = 9–10].

Twelve disease categories were found to be significantly related [−log(*P* value) = 4–14] only to Hcy-thiolactone (Fig. 3b): ‘lipid concentrations, risk of CAD’; ‘polygenic dyslipidemia’; ‘restenosis’; ‘deep vein thrombosis’; ‘stroke’; ‘myocardial infarction’; ‘aging’; ‘venous thromboembolism’; ‘placental abruption’; ‘inflammatory bowel disease’; ‘albuminuria’; ‘cardiac hypertrophy’; and ‘arterial hypertension.’

Seven disease categories were significantly [−log(*P* value) = 4–8] related only to *N*-Hcy-protein: ‘metabolic disease,’ ‘developmental diseases,’ ‘endometriosis,’ ‘albuminuria,’ ‘arterial hypertension,’ ‘DNA adducts,’ and ‘neurological diseases.’ However, none of these disease categories was associated only with Hcy (Fig. 3b).

### Network analysis

IPA identified a unique set of biological networks for each metabolite: eight for Hcy-thiolactone, three for *N*-Hcy-protein, and another three for Hcy. Two top-scored networks for Hcy-thiolactone (score = 34–40) were ‘cardiovascular disease, cardiac infarction, skeletal and muscular system development and function’ and ‘cardiovascular disease, lipid metabolism, small molecule biochemistry’ (Table 6). Two top-scored networks for *N*-Hcy-protein (score = 24–35) were ‘small molecule biochemistry, neurological disease’ and ‘cardiovascular system development and function’ (Table 6). Two top-scored networks for Hcy (score = 25–37) were ‘amino acid metabolism, lipid metabolism,’ ‘cellular movement, cardiovascular and nervous system development and function’ (Table 6).

**Table 6:** Top molecular networks of genes responding to Hcy-thiolactone, *N*-Hcy-protein, and Hcy

**Fig. 4:**
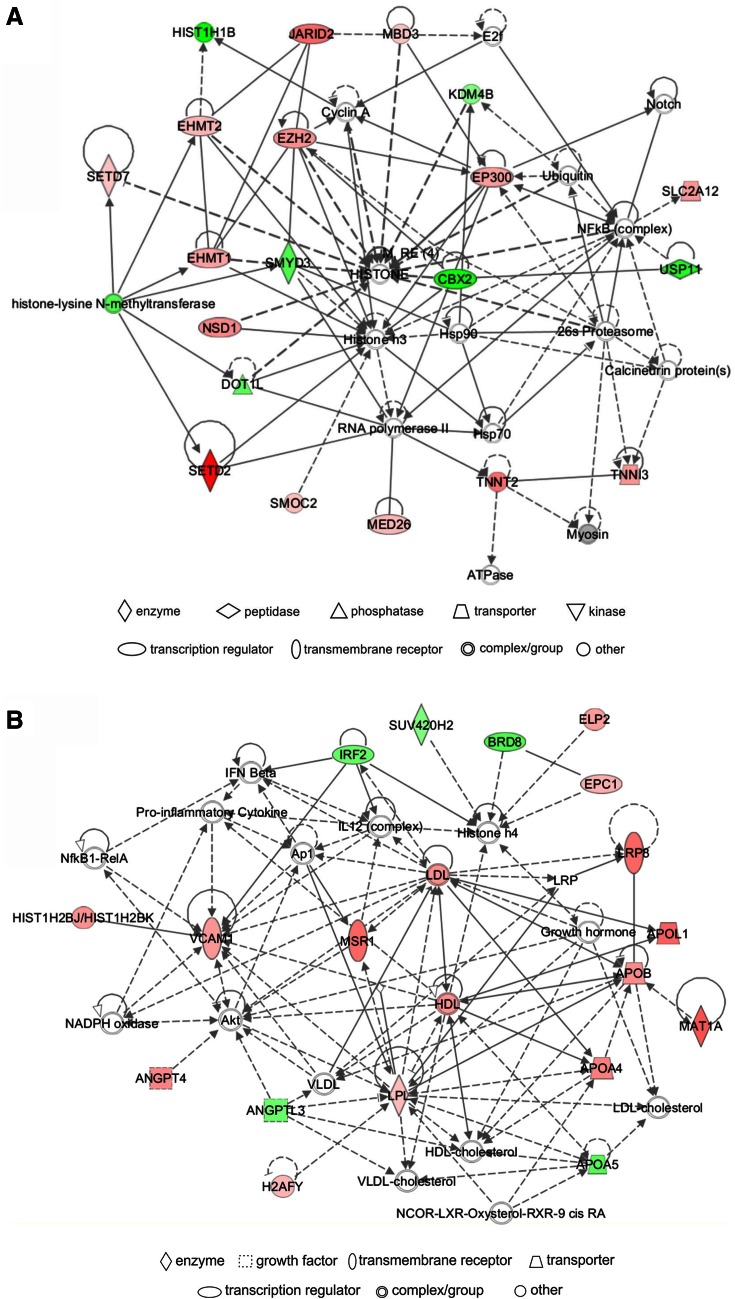
Top networks of Hcy-thiolactone-responsive genes. **a** Cardiovascular disease, cardiac infarction, skeletal and muscular system development and function network; **b** cardiovascular disease, lipid metabolism, small molecule biochemistry network. Direct (*solid lines*) and indirect (*dashed lines*) gene–gene interactions between genes up-regulated (*red*), down-regulated (*green*), and unaffected (*white*) by Hcy-thiolactone. The intensity of *green* and *red* node colors indicates the degree of *down*- or *up*-regulation, respectively. *Solid lines* indicate simple binding associations between protein products of gene expression, whereas *arrows* indicate that the gene at the arrowhead is being acted upon by the gene at the origin of the *arrow*

**Fig. 5:**
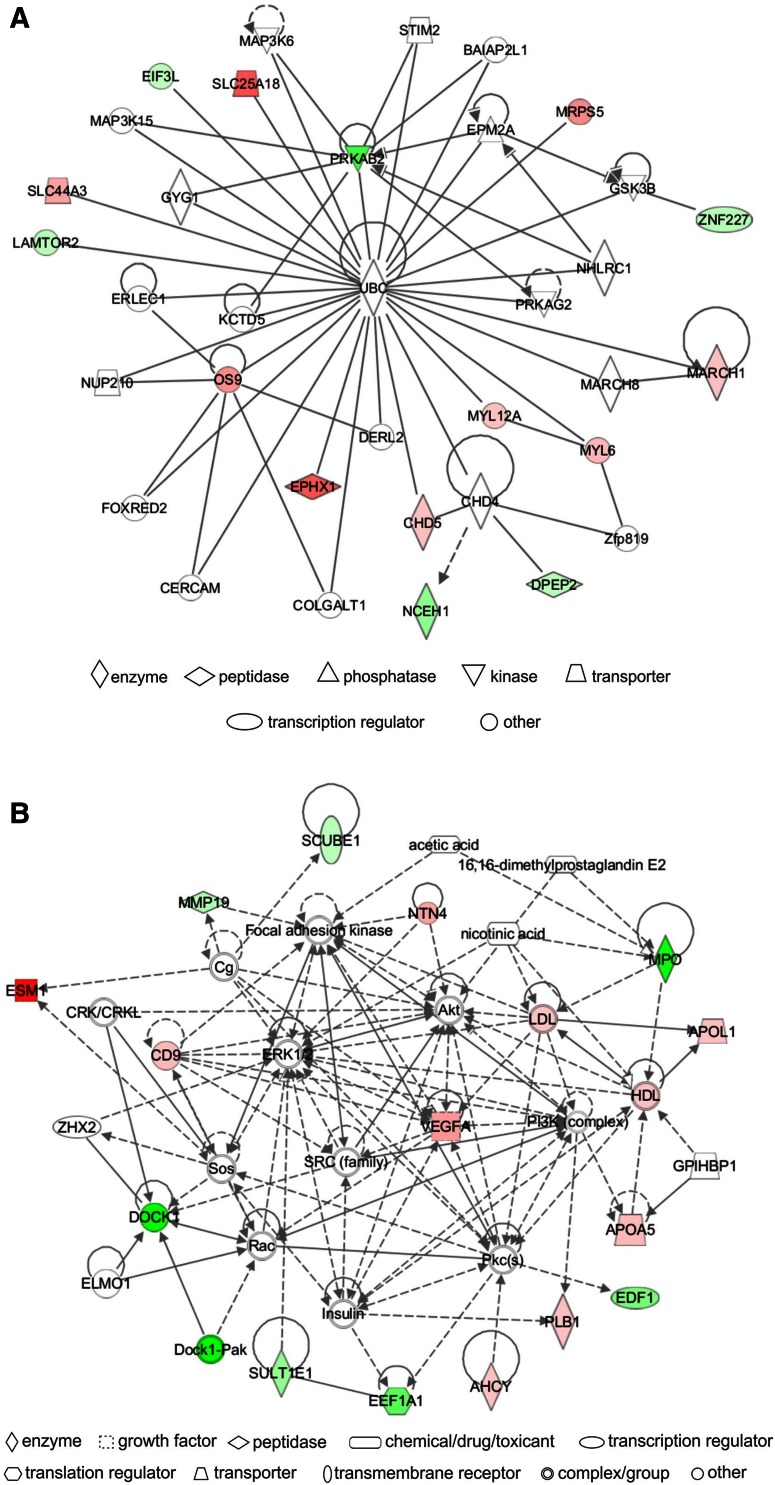
Top networks of *N*-Hcy-protein-responsive genes. **a** Small molecule biochemistry, neurological disease network; **b** cardiovascular system development and function, dermatological diseases and conditions network. Direct (*solid lines*) and indirect (*dashed lines*) gene–gene interactions between genes *up*-regulated (*red*), *down*-regulated (*green*), and unaffected (*white*) by *N*-Hcy-protein

**Fig. 6:**
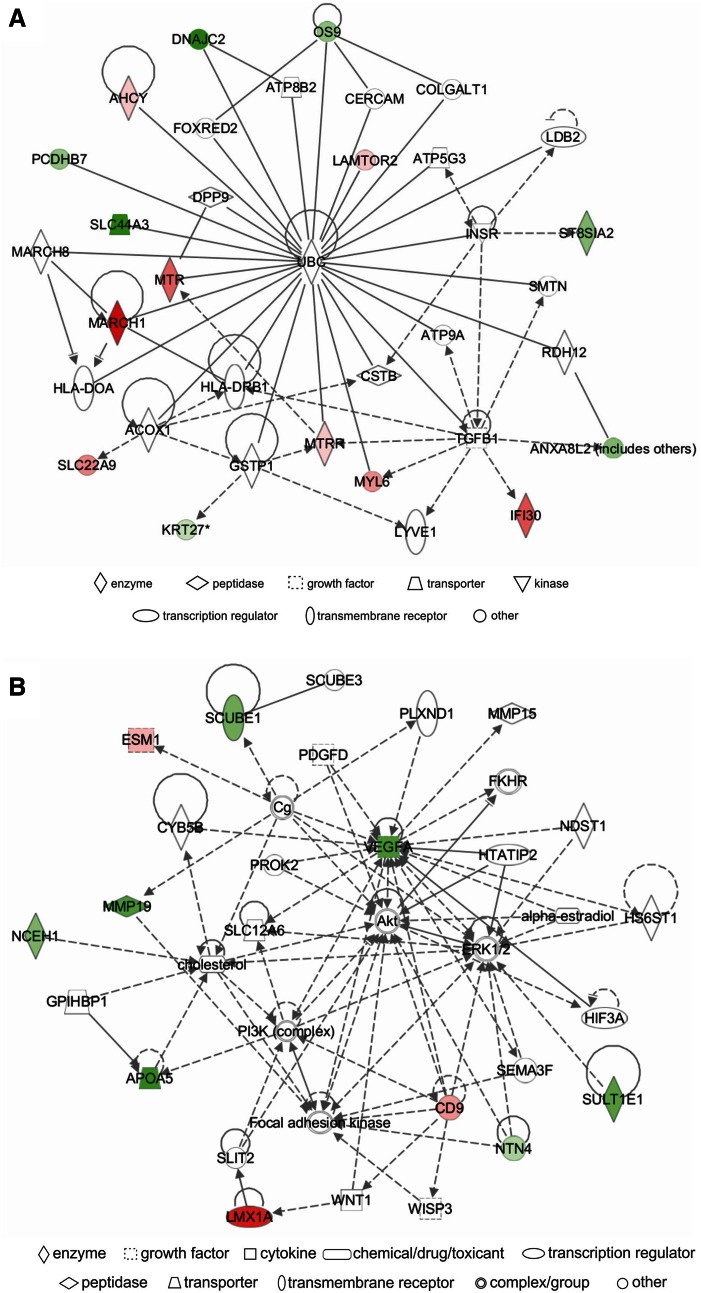
Top networks of Hcy-responsive genes. **a** Amino acid metabolism and lipid metabolism network; **b** cellular movement, cardiovascular system development and function, and nervous system development and function network. Direct (*solid lines*) and indirect (*dashed lines*) gene–gene interactions between genes *up*-regulated (*red*), *down*-regulated (*green*), and unaffected (*white*) by Hcy

Upregulated and downregulated genes are highlighter in red and green, respectively, Graphical representation of interactions between molecules in these networks is shown in Figs. 4, 5, and 6

A predominant network, with a score = 40, associated with Hcy-thiolactone contained 34 genes: 20 were quantified by microarrays and 14 were identified by IPA to interact with the quantified genes. Hcy-thiolactone exhibited strong interactions with histone/chromatin modification and transcription genes, endothelial cell growth, lipoprotein-related, and blood clotting genes (Fig. 4).

Different networks were associated with *N*-Hcy-protein and Hcy. The predominant *N*-Hcy-protein network, with a score = 35, contained 35 genes: 16 were identified by microarrays and 19 were identified by IPA to interact with the quantified genes (Fig. 5). This network reveals strong interaction of *N*-Hcy-protein with genes involved in cardiovascular system development and function.

The predominant Hcy network, with a score = 37, contained 35 genes: 16 were quantified by microarrays and 19 were identified by IPA to interact with the quantified genes. The IPA-identified network for genes regulated by Hcy revealed strong interactions of those genes with the ubiquitin C (UBC) posttranslational modification system that targets proteins for proteolytic degradation (Fig. 6).

## Discussion

Endothelial dysfunction plays a central role in CVD and is a common finding in HHcy in humans and animal models (Dayal and Lentz 2008; Lentz 2005). To understand the mechanisms by which HHcy disrupts normal cellular function and ultimately causes disease, we studied human endothelial cell transcriptome to identify genes whose expression is modulated by individual Hcy metabolites that accumulate in HHcy. We examined changes in gene expression induced by Hcy-thiolactone, *N*-Hcy-protein, and Hcy itself in HUVECs, a frequently used model of vascular cells (Jakubowski et al. 2000). We found that each metabolite affected the expression of unique sets of genes in different metabolic pathways and networks that are associated with CVD. Many of those genes and pathways were not previously associated with HHcy. Our results demonstrate that Hcy-thiolactone and *N*-Hcy-protein, rather than Hcy, cause changes in gene expression for the majority of genes.

### Pathways affected by Hcy-thiolactone

‘Chromatin organization’ was the top molecular pathway affected by Hcy-thiolactone (Fig. 3a). Out of 113 genes affected by Hcy-thiolactone, nineteen encoded chromatin proteins: histones, histone-modifying enzymes, and chromatin-binding proteins. These include histone-lysine methyltransferase genes *SEDT2*, *SEDT7*, *EZH2*, *EHMT1*, *EHMT2*, which were up-regulated, and *SUV420H2*, *DOT1L*, *SMYD3*, which were down-regulated. Histone-lysine acetyltransferase (*EPC1*, *EP300*) and ubiquitin transferase (*CBX2*) genes were up-regulated, while methyl-CpG-binding domain protein (*MBD3*) and lysine-specific demethylase (*JMJD2B*) genes were down-regulated. Another gene, encoding regulator of histone methylation protein Jumonji (*JARID2*), was up-regulated by Hcy-thiolactone. These findings suggest that epigenetic mechanisms involving histone modification might contribute to endothelial dysfunction induced by Hcy-thiolactone.

Posttranslational modifications of histone-lysine ε-amino groups by acetylation, methylation, and ubiquitination play a key role in the modulation of chromatin structure and DNA methylation status that govern epigenetic regulation of gene expression (Tessarz and Kouzarides 2014). Previous findings have shown that the status of DNA methylation can be affected by HHcy and that AdoHcy is responsible for epigenetic dysregulation of normal DNA methylation patterns associated with atherosclerosis (Handy et al. 2011; Ingrosso et al. 2003). However, other studies found that treatments with Hcy increase DNA methylation in human lymphocytes (Yi-Deng et al. 2007) and cardiomyocytes (Chaturvedi et al. 2014), suggesting that DNA methyltransferases are not inhibited by AdoHcy in those cells.

In mice, HHcy cause tissue-specific changes in H19 gene methylation and expression. In response to HHcy, H19 DNA methylation decreased in the liver but increased in the brain and aorta (Devlin et al. 2005). Furthermore, increased H19 DNA methylation resulted in a decrease in H19 expression in the brain but an increase in H19 expression was observed in the aorta. Levels of H19 transcripts in aorta correlated positively with plasma total Hcy (Devlin et al. 2005). These findings indicate that, in contrast to original suggestions (Castro et al. 2005; Jamaluddin et al. 2007), AdoHcy level may not be a major determinant of DNA methylation in HHcy and that other factors regulate DNA methylation and gene expression. Our present findings identify Hcy-thiolactone as a factor that can lead to epigenetic dysregulation of gene expression by affecting histones, histone-modifying enzymes, and other chromatin-associated proteins that control DNA methylation.

The involvement of a histone deacetylase and a methyl-CpG-binding protein, whose genes we found to be affected by Hcy-thiolactone, in the epigenetic modulation of gene expression by HHcy in vivo was documented in a mouse model in which HHcy was induced by subcutaneous administration of methionine (5.2 mmol/kg twice a day, for 7 days) (Dong et al. 2007). This treatment down-regulates reelin (RELN) expression in GABAergic interneurons by inducing hypermethylation of CpG islands in the *RELN* promoter in mouse brain. This process involves recruitment of transcription repressors methyl-CpG-binding protein (MeCP2) and histone deacetylases (HDAC) leading to the formation of transcriptionally inactive chromatin. Histone hyperacetylation induced by the administration of HDAC inhibitors leads to demethylation and activation of RELN promoter. DNA methyltransferase DNMT1, whose expression is not changed by Met treatment, is not responsible for RELN promoter demethylation (Dong et al. 2007).

‘One-carbon metabolism’ was the second top pathway affected by Hcy-thiolactone (Fig. 3a). We found that *MTHFD2* gene encoding mitochondrial bifunctional methylenetetrahydrofolate dehydrogenase/cyclohydrolase was up-regulated in HUVECs treated with Hcy-thiolactone, but not Hcy or *N*-Hcy-protein. Other investigators reported that *MTHFD2* is up-regulated in HUVECs treated with 10 mM Hcy, but not in cells treated with 1 mM Hcy (Kokame et al. 1996). However, because treatments of HUVECs with such high Hcy concentrations generate significant concentrations of Hcy-thiolactone in a reaction catalyzed by methionyl-tRNA synthetase (even in the presence of competing methionine) (Jakubowski 2002a; Jakubowski et al. 2000), our present results strongly suggest that the actual metabolite inducing MTHFD2 expression in the Kokame et al.’s experiments was Hcy-thiolactone rather than Hcy.

Three other genes of the ‘one-carbon metabolism’ pathway, *CBS*, *MTR*, and *MTRR*, that we found to be up-regulated by Hcy-thiolactone, *N*-Hcy-protein, and Hcy are known to be associated with CVD (Zhao et al. 2012). *ALDH1L1*, another gene of the ‘one-carbon metabolism’ pathway that we found to be up-regulated by Hcy-thiolactone, is associated with ischemic stroke (Williams et al. 2014). Since reduced activity of these genes causes disease, our findings suggest that their up-regulation induced by Hcy metabolites will facilitate Hcy metabolism and thus is protective. This conclusion is supported by findings from a genome-wide meta-analysis showing that *ALDH1L1* and *CBS* are strongly associated with the difference between pre- and post-methionine load test total Hcy levels (Williams et al. 2014).

Other top pathways affected by Hcy-thiolactone were related to lipid traits. Serum total cholesterol, low-density lipoprotein cholesterol, high-density lipoprotein cholesterol, and triglycerides are among the most important risk factors for CVD and are targets for therapeutic intervention. Of the several genes involved in ‘lipid localization,’ ‘lipoprotein metabolic processes,’ and ‘lipid and fatty acid transport’ pathways whose expression was affected by Hcy-thiolactone, four (*ANGPTL3*, *APOA1B*, *APOB*, *LPL*) are known to be associated with lipid traits (Teslovich et al. 2010). These findings are consistent with previous results showing that lipid metabolism is deregulated in HHcy (Maclean et al. 2012; Werstuck et al. 2001) and suggest that Hcy-thiolactone contributes to these disturbances of lipid homeostasis. We also found that macrophage scavenger receptor gene (*MSR1*) implicated in cholesterol deposition in arterial walls during atherogenesis (Gough et al. 1998) was up-regulated by Hcy-thiolactone. This finding suggests a possible mechanistic link between HHcy and atherogenesis.

We also found that two genes encoding protein components of the mTOR signalling pathway were up-regulated by Hcy-thiolactone: the ragulator complex protein LAMTOR2 that recruits mTORC1 to lysosomes (Nada et al. 2009) and the DNA-damage-inducible transcript 4-like protein DDIT4L, a negative regulator of signal transduction that inhibits cell growth by regulating the TOR signaling pathway upstream of the TSC1–TSC2 complex (Corradetti et al. 2005). Amino acid sensing by mTORC1 involves leucyl-tRNA synthetase (Han et al. 2012), which is also known to metabolize Hcy to Hcy-thiolactone (Jakubowski 1995, 2011, 2012, 2013; Sikora and Jakubowski 2009). Our findings that *LAMTOR2* expression is down-regulated by *N*-Hcy-protein and up-regulated by Hcy (Fig. 1c) highlight the unique role of each Hcy metabolite in mTOR signalling. Up-regulation of DDIT4L that we observe in endothelial cells in response to Hcy-thiolactone is also observed in atherosclerotic lesions relative to healthy segments of the same artery (Cuaz-Perolin et al. 2004). Since protein *N*-homocysteinylation by Hcy-thiolactone occurs in atherosclerotic lesions, as shown in *ApoE*-/- mouse model (Perla-Kajan et al. 2008), these findings provide further support for a pro-atherogenic role of Hcy-thiolactone.

### Pathways affected by Hcy-thiolactone, *N*-Hcy-protein, and Hcy

Our finding that the blood coagulation pathway is affected by Hcy-thiolactone, *N*-Hcy-protein, and Hcy in a metabolite-specific manner is consistent with a pro-thrombotic phenotype of HHcy patients and suggests that each metabolite contributes to thrombosis. Severe HHcy is associated with thromboembolism, the major cause of morbidity and mortality in homocystinuric patients (Mudd et al. 1985). Mild HHcy is also a risk factor for venous thrombosis in the general population (Den Heijer et al. 2005). Previous findings demonstrated the involvement of Hcy-thiolactone and Hcy in two distinct mechanisms that may contribute to pro-thrombotic phenotype associated with HHcy. One mechanism involves the modification of fibrinogen (Fbg) by Hcy-thiolactone, which generates *N*-Hcy-Fbg that forms fibrin clots resistant to lysis by plasmin (Sauls et al. 2006). Hcy-thiolactone and *N*-Hcy-protein, including pro-thrombotic *N*-Hcy-Fbg (Sauls et al. 2006, 2011), are elevated in homocystinuric patients (Jakubowski et al. 2008). One of the three *N*-Hcy-lysine residues in Fbg from homocystinuric patients, *N*-Hcy-Lys562, is located in an unstructured region (α392-610) of the α-chain known to be involved in tPA and plasminogen binding, which can explain the abnormal characteristics of fibrin clots formed from *N*-Hcy-Fbg (Jakubowski 2013; Sikora et al. 2014). In another mechanism, based on studies in mice with dietary HHcy, Hcy was shown to form a disulfide linkage with Cys9 residue of annexin A2, which reduces perivascular thrombolytic activity by preventing the cell surface-dependent plasmin generation (Jacovina et al. 2009).

Our present findings showing that the blood coagulation pathway is affected by Hcy-thiolactone, *N*-Hcy-protein, and Hcy suggest that, in addition to direct effects on the components of blood clotting system such as Fbg and annexin A2, Hcy-thiolactone and Hcy may affect vascular homeostasis by changing the expression of genes encoding proteins involved in blood clotting (*CD9*, *ANXA8*, *SCUBE1* for Hcy-thiolactone and Hcy; *VWF*, *FGB*, *MMRN1*, *PLG*, *LRP8* for Hcy-thiolactone). Furthermore, some blood coagulation genes (*CD9*, *ANXA8*, *SCUBE1*) may also be affected by *N*-Hcy-protein. Taken together, these findings suggest that each of the three metabolites exerts a specific effect on vascular homeostasis. In addition, elevated expression of VWF in response to Hcy-thiolactone may contribute to increased restenosis observed in cardiac surgery patients with HHcy (Schnyder et al. 2001).

We found that *MTR*, *MTRR*, *AHCY*, and *CBS* genes encoding enzymes involved in sulfur amino acid metabolism were up-regulated by Hcy-thiolactone, *N*-Hcy-protein, and Hcy. Of these, low activity *CBS*, *MTR*, and *MTRR* variants are known to be associated with CVD (Zhao et al. 2012). These findings suggest a protective mechanism in which the accumulation of toxic Hcy metabolites such as Hcy-thiolactone and *N*-Hcy-protein up-regulates the expression of genes involved in the removal of Hcy via trans-methylation and trans-sulfuration pathways that prevent the accumulation of these toxic metabolites. That CBS is important for maintaining endothelial cell homeostasis is supported by findings showing that reduction in CBS expression induces premature senescence in HUVECs (Albertini et al. 2012).

Highly significant molecular pathways affected by Hcy-thiolactone, *N*-Hcy-protein, and Hcy include the ‘lipid, fatty acid, and steroid metabolism’ pathway. Fifteen genes of this pathway were affected by Hcy-thiolactone, seven genes were affected by *N*-Hcy-protein, and four by Hcy. Of these, three genes (*ANXA8*, *APOA5*, *ST8SIA2*) were affected by each of the three metabolites, and one gene (*SULT1E1*) was affected by *N*-Hcy-protein and Hcy. These findings are consistent with lipid traits associated with HHcy (Maclean et al. 2012; Werstuck et al. 2001) and suggest that each of the three metabolites—Hcy-thiolactone, *N*-Hcy-protein, and Hcy—may contribute to the modification of lipid traits in a metabolite-specific manner.

### Diseases related to Hcy-thiolactone, *N*-Hcy-protein, and Hcy

Bioinformatic analyses show that most of the disease categories related to Hcy-thiolactone, *N*-Hcy-protein, and Hcy involve the cardiovascular and central nervous systems. These diseases are known from previous studies to be associated with HHcy. However, our findings indicate that a specific Hcy metabolite is associated with a specific disease. Surprisingly, the ‘cardiovascular disease’ category was associated only with Hcy-thiolactone and *N*-Hcy-protein, but not with Hcy. Furthermore, while some diseases were associated only with Hcy-thiolactone or *N*-Hcy-protein, none was associated only with Hcy. Instead, diseases associated with Hcy were also associated with Hcy-thiolactone and *N*-Hcy-protein. For example, we found that Hcy-thiolactone was related to coronary artery disease, dyslipidemia, deep vein thrombosis, stroke, myocardial infarction, venous thromboembolism, placental abruption, preeclampsia, cardiac hypertrophy, and neurological disease. These findings are consistent with clinical manifestations of severe HHcy in genetic deficiencies of Hcy metabolism (Mudd et al. 2001; Strauss et al. 2007) and in mild HHcy in the general population (Joseph and Loscalzo 2013; Refsum et al. 2006) and suggest that Hcy-thiolactone might play a predominant role in these diseases. Another metabolite, *N*-Hcy-protein, might play a predominant role in developmental and neurological diseases.

We have also found that Hcy-thiolactone and *N*-Hcy-protein, in addition to Hcy, are linked to other diseases including cerebrovascular disease, ischemic disease, congenital heart disease, Alzheimer’s disease, and neural tube defects. Taken together, these findings suggest that each Hcy metabolite might have a specific disease-related role.

Our findings suggest that cancer is related to Hcy-thiolactone, *N*-Hcy-protein, and Hcy (Fig. 3b). Previous studies have associated cancer with HHcy (Plazar and Jurdana 2010), and our present findings suggest that each of the three metabolites can contribute. Hcy metabolism is known to be deregulated in cancer cells, which in contrast to normal cells cannot grow on Hcy-containing media (Fiskerstrand et al. 1994). Hcy-thiolactone and *N*-Hcy protein are elevated in cancer cells, which likely contributes to the growth defect on Hcy media (Jakubowski 1997a, b; Jakubowski and Goldman 1993). Deregulation of central carbon metabolic pathways has long been linked to cancer as discussed in a recent hypothesis (Konieczna et al. 2015), and we now show that changes in gene expression induced by Hcy-thiolactone, *N*-Hcy-protein, and Hcy can also be involved.

The IPA analysis of pathways and networks based on gene expression results shows that Hcy-thiolactone strongly relates to ‘CVD, cardiac infarction, skeletal, and muscular systems’ among others (Table 6; Fig. 4). This is in line with the commonly reported manifestations of the HHcy and suggests the involvement of Hcy-thiolactone in those pathologies. This network (Table 6; Fig. 4) contains genes involved in chromatin organization/modification, which suggests important role of those genes in heart pathology induced by HHcy. Top network identified for *N*-Hcy-protein involves genes affecting ‘cardiovascular system development and function, dermatological disease, and conditions’ (Table 6; Fig. 5), which suggests that *N*-Hcy-protein contributes to heart and skin pathologies observed in HHcy. Top network identified for Hcy involves genes affecting ‘amino acid and lipid metabolism’ (Table 6; Fig. 6). This is consistent with previous studies that demonstrated the deregulation of amino acid (Akahoshi et al. 2014) and lipid (Maclean et al. 2012; Werstuck et al. 2001) metabolism in HHcy in mouse models.

HHcy is known to cause pathology in essentially all organs, including the cardiovascular and central nervous systems (Mudd et al. 2001). These pathologies are initiated by endothelial cell dysfunction and damage (Dayal and Lentz 2008). Thus, it may not be unexpected that exposure of HUVECs to individual metabolites such as *N*-Hcy-protein, Hcy-thiolactone, and Hcy affects the expression of genes involved in neurological disease, ischemic disease, congenital heart disease, Alzheimer’s disease, and neural tube defects.

In conclusion, our findings represent the first direct evidence that each Hcy metabolite uniquely modulates gene expression in molecular pathways and biological networks important for vascular homeostasis and identifies new genes and metabolic pathways linked to CVD. We also demonstrate that Hcy-thiolactone and *N*-Hcy-protein, rather than Hcy, affect the majority of genes. One of the pathways containing genes affected only by Hcy-thiolactone is involved in chromatin/histone modifications. Thus, our findings suggest that alterations in chromatin modification gene expression might contribute to endothelial dysfunction and provide a possible mechanistic link between each metabolite and atherothrombosis. In addition, our findings underscore the importance of examining histone modifications in the context of HHcy-induced pathologies in humans.

## Abbreviations

CBS: Cystathionine β-synthase
CVD: Cardiovascular disease
Fbg: Fibrinogen
Hcy: Homocysteine
HHcy: Hyperhomocysteinemia
HUVECs: Human umbilical vein endothelial cells
IPA: Ingenuity pathway analysis
RT-qPCR: Real-time quantitative polymerase chain reaction
